# Supplementary material for: Single-Cell RNA Sequencing of Donor-Reactive T Cells Reveals Role of Apoptosis in Donor-Specific Hyporesponsiveness of Kidney Transplant Recipients
Source: Int J Mol Sci. 2023 Sep 23;24(19):14463. doi: 10.3390/ijms241914463 (PMC10572284; doi:10.3390/ijms241914463)
Supplement: Supplementary file 1 [file ijms-24-14463-s001.zip › 230812_vanderList et al_SupportingInfo.pdf]

Supporting Information

Supporting Information Table of contents

Supplementary Tables

Table S1: Stable kidney transplant recipient characteristics

Table S2: Antibody panel for donor-reactive T cell sorting

Table S3: Cell numbers and reads from TCR and transcriptome sequencing

Table S4: Differential expression analysis to define the cell identity of each cluster (filtered for adjusted p-value of <0.0001 and selected top 20 based on absolute logFC)

Table S5: Gene Ontology Enrichment Results (top 5 up and top 5 down) differentially expressed genes (p adjusted value <0.01) between clusters

Table S6: Genes with differential expression (adjusted p-value < 0.01) between third-party-stimulated alloreactive T cells within clusters from before to 3-5 years after transplant

Table S7: Gene Ontology Enrichment Results (top 10 up and top 10 down) based on differentially expressed genes (adjusted p-value <0.1) between third-party samples from a pre and post timepoint per cluster

Table S8: Number of cells with a TRA or TRB clonotype shared between pre and post timepoint samples of the same patient (P0, P1, P2) per cluster

Supplementary Figures

Figure S1: Flow cytometric gating strategy for sorting viable donor-reactive T cells.

Figure S2: Quality and filtering of sequenced donor-reactive T cells.

Figure S3: Clustering tree obtained through clustree package.

Figure S4: Number of reads for TRA, TRB and TRD clonotypes of sequenced donor-reactive T cells.

Figure S5: Expression of (non)-T cell markers validating sequencing of donor-reactive (CD3+CD137+) T cells.

Figure S6: Expression of T cell subset markers (naïve, memory).

Supplementary Figures

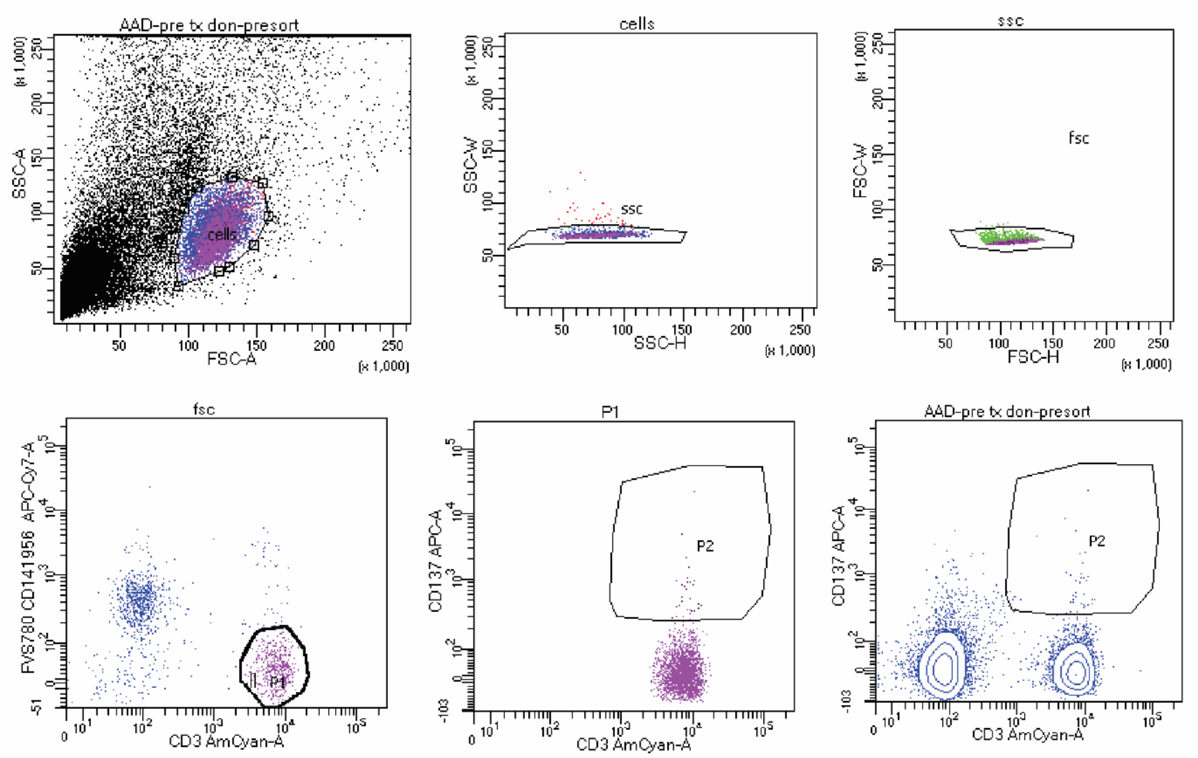

Tube: presort

Population	#Events	%Parent	%Total
All Events	43,981	####	100.0
cells	6,929	15.8	15.8
ssc	6,729	97.1	15.3
fsc	6,722	99.9	15.3
P1	2,380	35.4	5.4
P2	31	1.3	0.1

Figure S1: Flow cytometric gating strategy for sorting viable donor-reactive T cells. Flow cytometric images from a representative donor-stimulated sample from prior to transplantation depicts gating for CD137+CD3+DUMP- cells. First, total lymphocytes are gated with SSC-A and FSC-A followed by gating for single cells using SSC-W and SSC-H. Total T cells are gated from single lymphocytes for CD3 expression while gating out cells positive for unwanted (DUMP) cells; FVS780, CD14, CD19, and CD56. Finally, CD137 expression is used to gate on viable CD3+ T cells for the total donor-reactive (CD137+) T cell population.

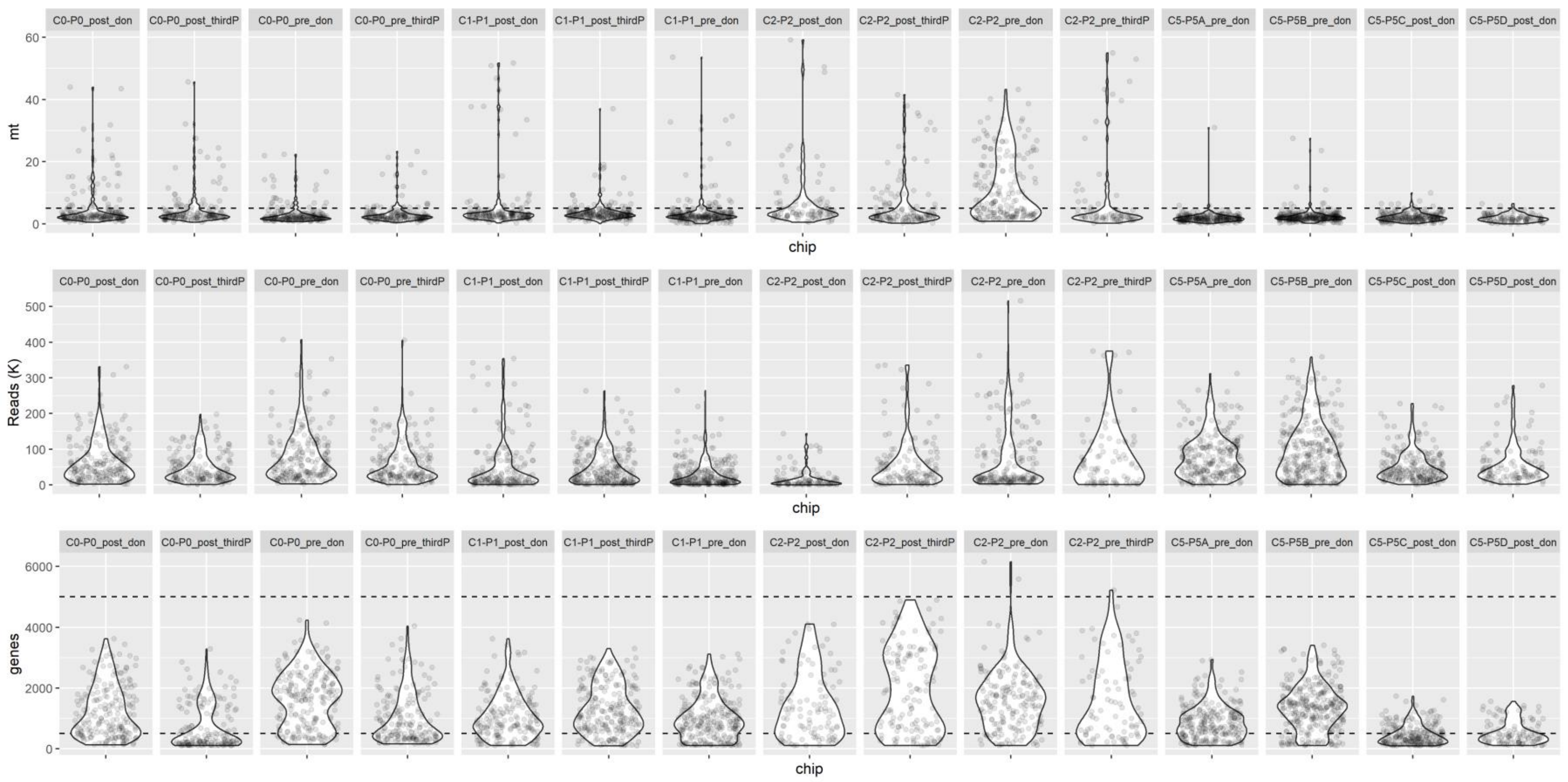
C0-P0_post_don
C0-P0_post_thirdP
C0-P0_pre_don
C0-P0_pre_thirdP
C1-P1_post_don
C1-P1_post_thirdP
C1-P1_pre_don
C2-P2_post_don
C2-P2_post_thirdP
C2-P2_pre_don
C2-P2_pre_thirdP
C5-P5A_pre_don
C5-P5B_pre_don
C5-P5C_post_don
C5-P5D_post_don
mt
0
20
40
60
chip
Reads (K)
0
100
200
300
400
500
genes
0
2000
4000
6000

Figure S2: Quality and filtering of sequenced donor-reactive T cells. The number of genes, total reads (K) and reads for mitochondrial genes (mt) detected (depicted on the Y-axis) for each single cell plotted per sample (depicted on the X-axis).

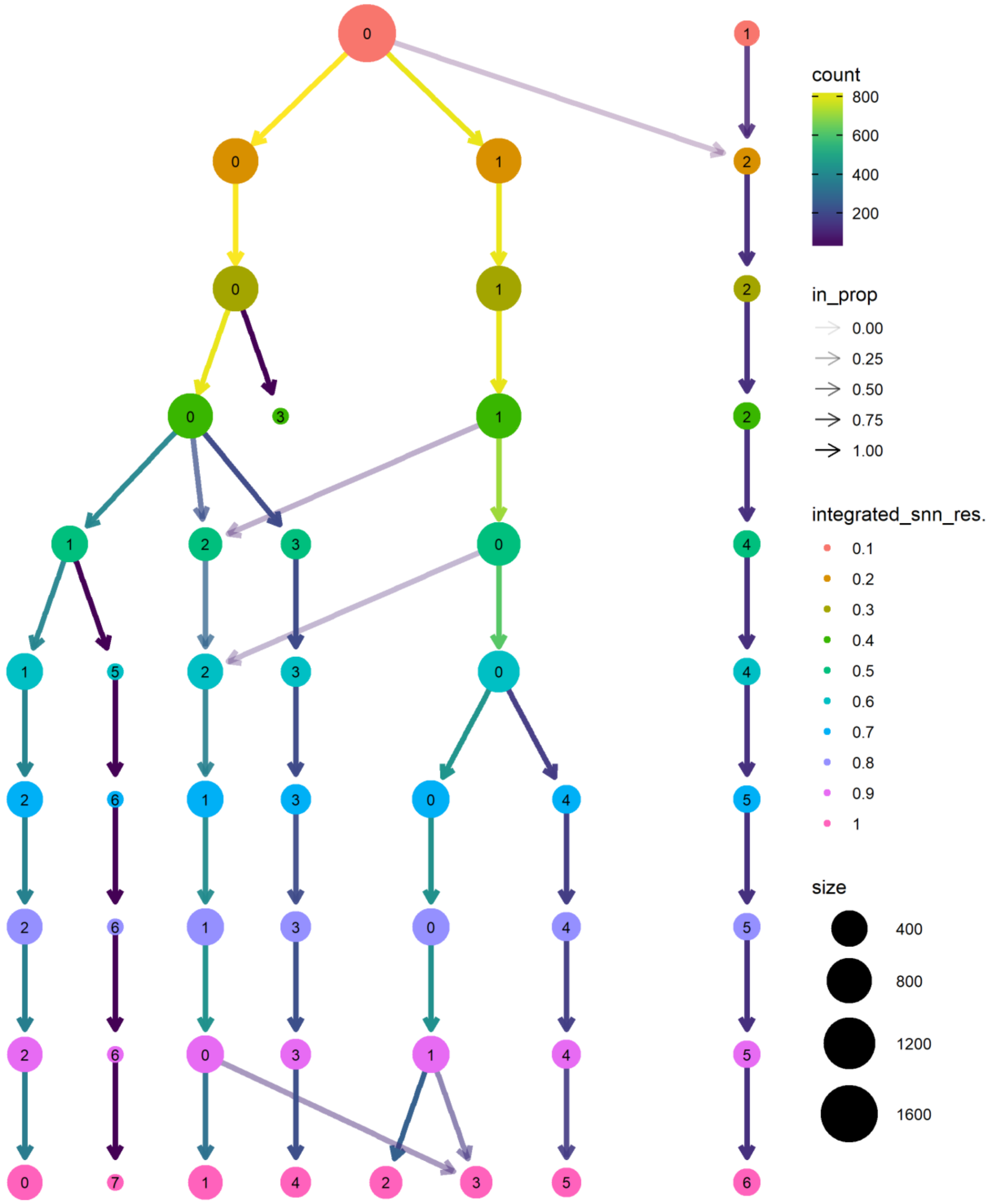

Figure S3: Clustering tree obtained through clustree package. Nodes are coloured according to the resolution and sized according to the number of cells they contain. Arrows are coloured according to the number of cells (from blue around 200 cells to yellow representing around 800). The transparency is adjusted according to the in-proportion, with stronger lines showing arrows that are more important to the higher-resolution cluster.

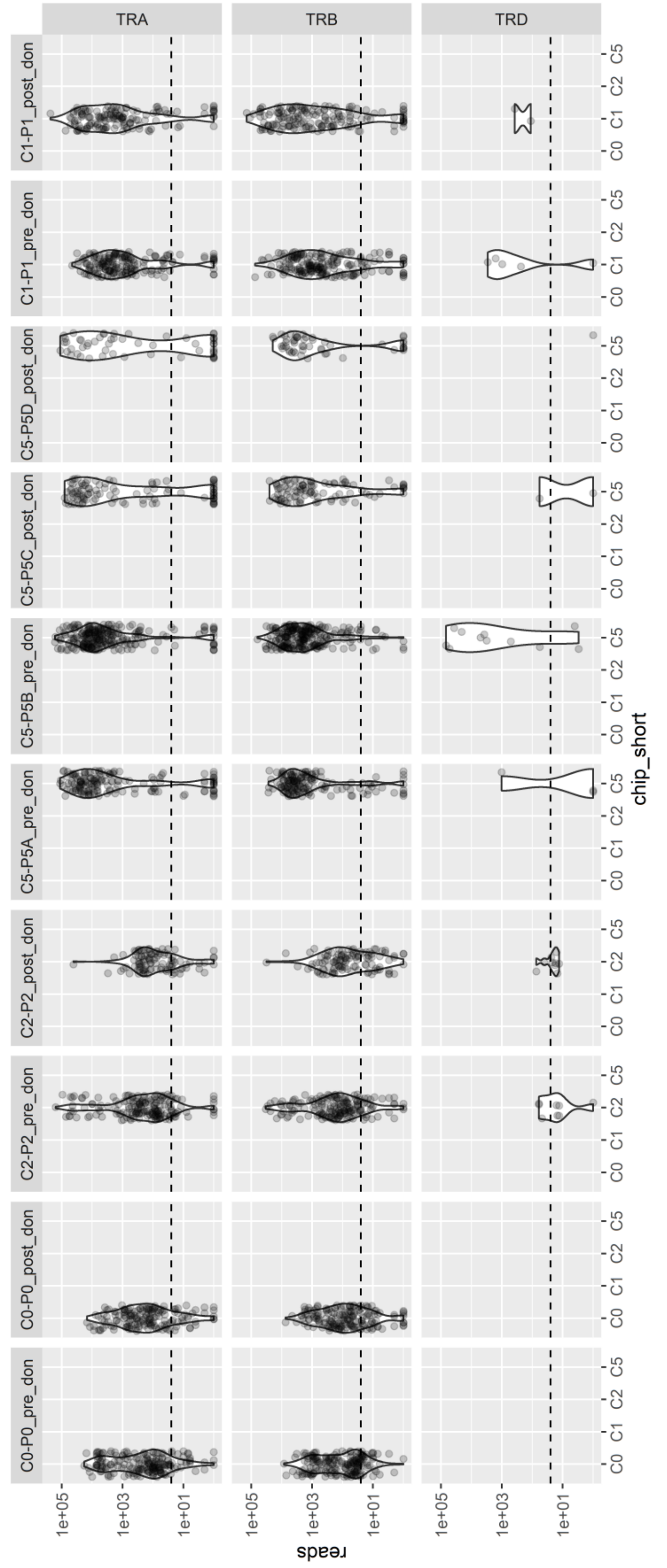

Figure S4: Number of reads for TRA, TRB and TRD clonotypes of sequenced donor-reactive T cells. The total number of reads (depicted on the Y-axis) for each single cell plotted per sample (depicted on the X-axis).

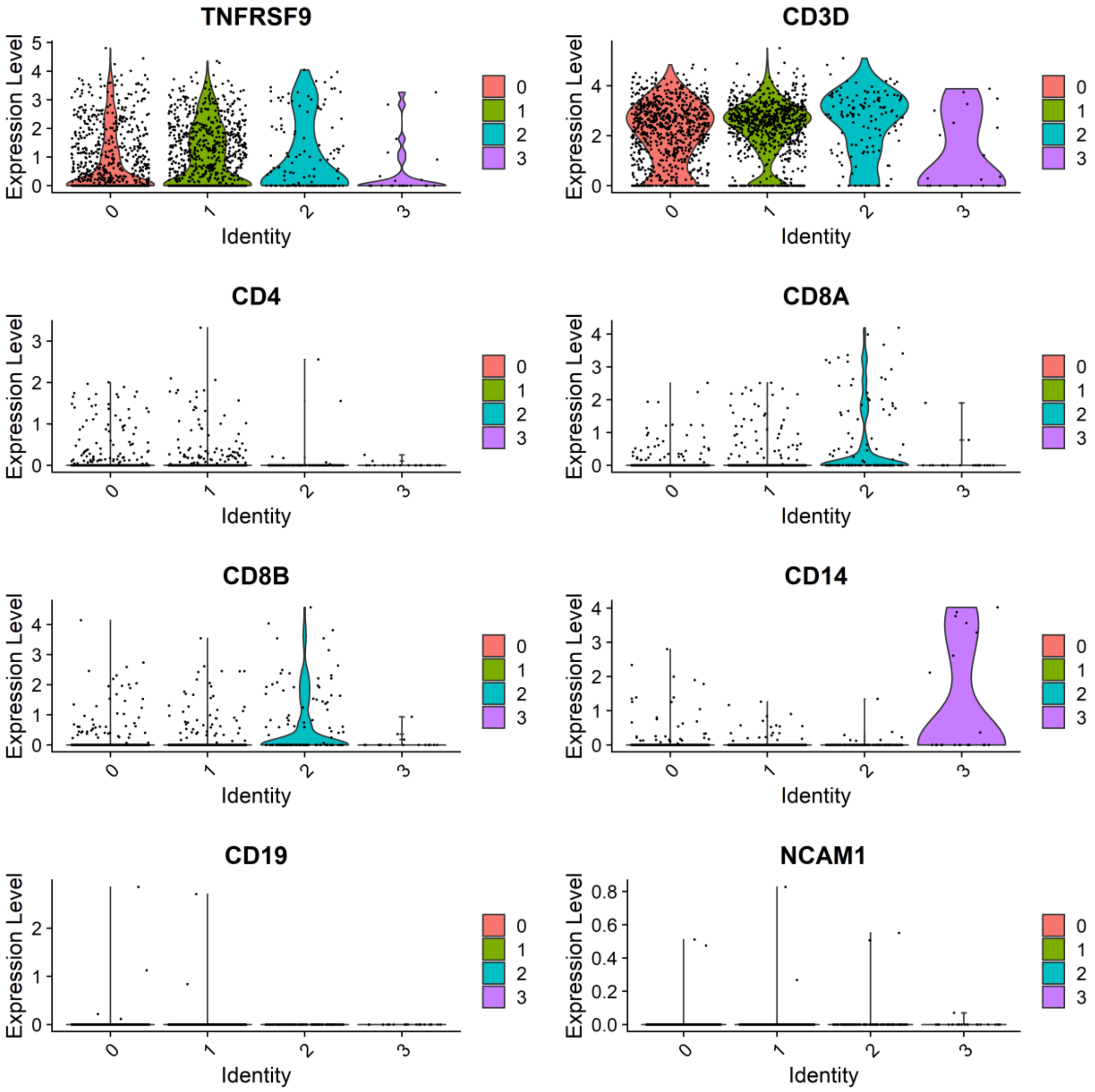

Figure S5: Expression of (non)-T cell markers validating sequencing of donor-reactive (CD3+CD137+) T cells. Violin plots showing the expression levels per cell within each cluster of CD137 gene (*TNFRSF9*), the CD3 subunit gene *CD3D*, as well as *CD4*, *CD8A* and *CD8B*. Genes expressed monocytes, B-cells and natural killer cells (*CD14*, *CD19*, *NCAM1*, respectively) are also depicted.

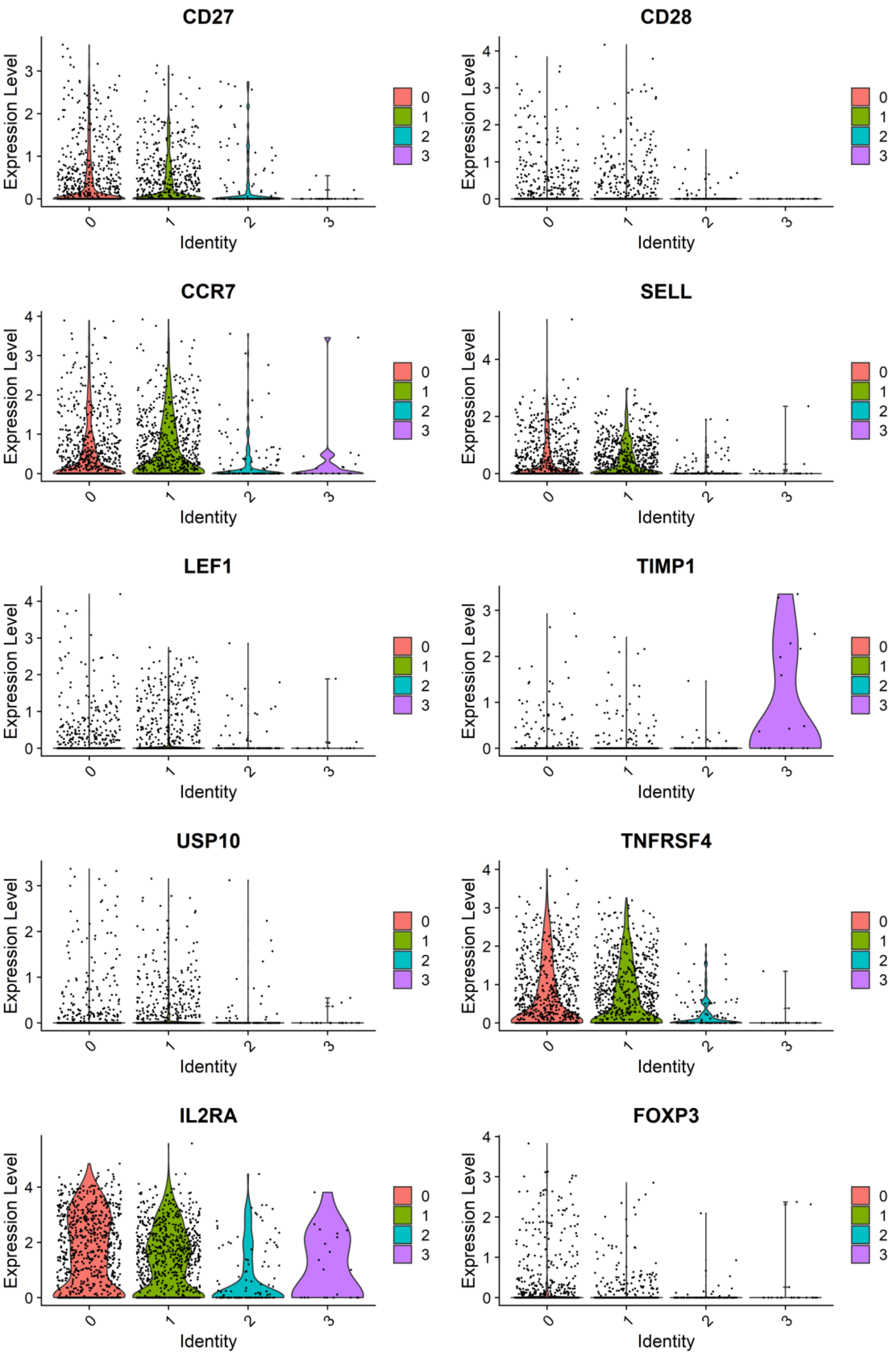

Figure S6: Expression of T cell subset markers (naïve, memory). Violin plots showing the expression levels per cell within each cluster of T cell genes used to identify T cell subsets. Common genes used to distinguish differentiation of T cells include *CD27* and *CD28*. According to a study by Wang et al. (2022) the following genes are used to identify CD4 naive

T cells (*CCR7*, *LEF1*, *SELL*), CD4 memory T cells (*TNFRSF4, TIMP1, USP10*) and CD4 Tregs (*FOXP3, IL2RA*).
